# Multivariate genomic analyses of frailty reveal the unique importance of cognitive and multimorbidity pathways for aging‐related health outcomes

**DOI:** 10.1002/alz.087481

**Published:** 2025-01-09

**Authors:** Isabelle F Foote, Jonny P Flint, John D Fisk, Tobias K Karakach, Andrew Rutenberg, Nicholas G Martin, Michelle K Lupton, Simon R Cox, Michelle Luciano, Kenneth Rockwood, Andrew D Grotzinger

**Affiliations:** ^1^ University of Colorado Boulder, Boulder, CO USA; ^2^ Queen Mary University of London, London, England United Kingdom; ^3^ University of Edinburgh, Edinburgh, Scotland United Kingdom; ^4^ Lothian Birth Cohort Studies, Edinburgh, Scotland United Kingdom; ^5^ Dalhousie University, Halifax, NS Canada; ^6^ Nova Scotia Health, Halifax, NS Canada; ^7^ QIMR Berghofer Medical Research Institute, Brisbane, QLD Australia

## Abstract

**Background:**

Frailty is a complex clinical state that is associated with poorer health outcomes and increased dementia risk in older adults. It is routinely measured using the Frailty Index, which is a proportional score based on the number of ‘deficits’ that an individual has. Whilst such measures are useful for risk assessment, the aggregation of highly heterogeneous deficit profiles in genetic studies may obscure important insights into the underlying biology of frailty.

**Method:**

We used Genomic Structural Equation Modelling to model shared genetic signal between 30 deficits from the Frailty Index using summary data from genome‐wide association studies (GWAS). This involved conducting a multivariate GWAS to identify genomic risk loci associated with each of the latent frailty factors in our model. We used polygenic risk scores (PRS) to test the predictive accuracy of the latent factors for detecting frailty status in 3 external cohort studies. Finally, we tested the genetic correlation between our latent frailty factors and >50 aging‐related outcomes, including dementia.

**Result:**

The genetic architecture of the frailty deficits was best captured by a general factor influencing all 30 deficits, plus 6 additional latent factors representing genetic overlap between distinct subsets of deficits that were uncorrelated with the general factor. These 6 factors can be broadly described as reflecting pathways linked to social isolation, unhealthy lifestyle, multimorbidity, metabolic/respiratory status, cognition, and disability. GWAS conducted to measure the effects of individual genetic variants on these frailty factors identified 408 genomic risk loci. Our PRS analyses demonstrated consistent evidence that shared genetic pathways between frailty deficits linked to multimorbidity and cognition, but unique of the general frailty factor, are particularly important for predicting frailty status. Genetic correlation analyses further highlighted a significant genetic association between frailty pathways linked to cognition and Alzheimer’s disease (*rg* = 0.32, SE = 0.07, *p* = 5.58×10^−06^).

**Conclusion:**

Our findings provide novel insights into the characterization of general and specific pathways within the frailty state at the genetic level. We demonstrate how refining our knowledge of frailty, particularly in relation to multimorbidity and cognitive impairment, may help to stratify frail individuals at increased risk of developing dementia or other adverse health outcomes.

